# Sevoflurane Induces Endoplasmic Reticulum Stress Mediated Apoptosis in Hippocampal Neurons of Aging Rats

**DOI:** 10.1371/journal.pone.0057870

**Published:** 2013-02-28

**Authors:** Gang Chen, Ming Gong, Min Yan, Xiaoming Zhang

**Affiliations:** 1 Department of Anesthesiology, the Second Affiliated Hospital, School of Medicine, Zhejiang University, Hangzhou, China; 2 Department of Anatomy and Cell Biology, School of Medicine, Zhejiang University, Hangzhou, China; Università Vita-Salute San Raffaele, Italy

## Abstract

Elderly patients are more likely to suffer from postoperative memory impairment for volatile anesthetics could induce aging neurons degeneration and apoptosis while the mechanism was still elusive. Therefore we hypothesized that ER stress mediated hippocampal neurons apoptosis might play an important role in the mechanism of sevoflurane-induced cognitive impairment in aged rats. Thirty 18-month-old male Sprague-Dawley rats were divided into two groups: the sham anesthesia group (exposure to simply humidified 30–50% O_2_ balanced by N_2_ in an acrylic anesthetizing chamber for 5 hours) and the sevoflurane anesthesia group (received 2% sevoflurane in the same humidified mixed air in an identical chamber for the same time). Spatial memory of rats was assayed by the Morris water maze test. The ultrastructure of the hippocampus was observed by transmission electron microscopy (TEM). The expressions of C/EBP homologous protein (CHOP) and caspase-12 in the hippocampus were observed by immunohistochemistry and real-time PCR analysis. The apoptosis neurons were also assessed by TUNEL assay. The Morris water maze test showed that sevoflurane anesthesia induced spatial memory impairment in aging rats (*P*<0.05). The apoptotic neurons were condensed and had clumped chromatin with fragmentation of the nuclear membrane, verifying apoptotic degeneration in the sevoflurane group rats by TEM observation. The expressions of CHOP and caspase-12 increased, and the number of TUNEL positive cells of the hippocampus also increased in the sevoflurane group rats (*P*<0.05). The present results suggested that the long time exposure of sevoflurane could induce neuronal degeneration and cognitive impairment in aging rats. The ER stress mediated neurons apoptosis may play a role in the sevoflurane-induced memory impairment in aging rats.

## Introduction

There is extensive literature reporting that elderly patients are more likely than young patients to suffer from postoperative memory and cognitive deficits [1]. Evidences from animal models suggest that general anesthesia with volatile anesthetics alone produces impairment in learning and memory that persists for weeks or months [2], [3], [4]. Potential mechanisms for postoperative memory impairment are still elusive, such as brain cell apoptosis, accumulation of amyloid deposits in aging brain and suppression of synaptic transmission [4], [5], [6], [7]. Endoplasmic reticulum (ER) has several important functions, involving synthesis, folding, and posttranslational modification of secretory and membrane proteins [8]. ER stress under any harmful conditions could lead to decrease protein synthesis and increase the expression of molecular chaperones that promote proper folding and cellular recovery [9]. However, prolonged activation of ER stress ultimately initiates the apoptotic pathway [10]. Several proteins have been implicated in this apoptotic pathway, including a transcription factor, C/EBP homologous protein (CHOP), and the ER-resident caspase, caspase-12 [11], [12]. Increasing evidences demonstrated that ER stress may play a critical role in the pathogenesis of many acute and chronic neurodegenerative disorders, such as cerebral ischemia, Alzheimer’s disease, Parkinson’s disease and amyotrophic lateral sclerosis [10], [13], [14].

Volatile anesthetics could induce neuronal degeneration and apoptosis, then lead to memory impairment [15], [16], [17]. Evidences from animal models suggested volatile anesthetics interaction with neurodegenerative mechanisms, such as the onset and progression of Alzheimer’s disease [5], [16], [17]. Sevoflurane is one of the most commonly used volatile anesthetics and it had been shown to impair long-term emotional memory consolidation in human research [18], and especially in old mice [19]. But the underlying molecular mechanisms are still unknown. Therefore, we hypothesized that ER stress mediated hippocampal neurons apoptosis then induced neurons lost in the aging brain under long time sevoflurane exposure. It might play a role in the sevoflurane-induced memory impairment in aging rats.

## Methods

### Ethics Statement

This study was approved by the Institutional Animal Care and Use Committee of Zhejiang University under the NIH Guide for the Care and Use of Laboratory Animals.

### Animals and Anesthesia Exposure

Thirty 18-month-old male Sprague-Dawley rats (550 to 750 g) were used for this study. These rats were allowed to acclimate in the animal care facility for 1 week before the experiment, with free access to food and water, and maintained on a 12 h/12 h light/dark cycle. These rats were randomly divided into two groups (n = 15): the sham anesthesia (control group) and the sevoflurane anesthesia (sevoflurane group).

The sham anesthesia rats were exposed to simply humidify 30–50% O_2_ balanced by N_2_ in an acrylic anesthetizing chamber for 5 hours, while the sevoflurane anesthesia rats received 2% sevoflurane in the same mixed air in an identical chamber for 5 hours. In our preliminary experiment, five aging rats were exposed to 2% sevoflurane for 5 hours in the above mentioned anesthetizing chamber. These rats were not used for any other part of the study. A 24-g polyethylene catheter inserted into the tail artery after induction of sevoflurane anesthesia for hourly blood gas analysis and invasive blood pressure measurements. The blood sample was withdrawn every hour from the tail channel to determine the blood pH, arterial carbon dioxide tension, arterial oxygen tension, base excess, and blood glucose with a blood gas Analyzer. The results of preliminary experiment indicated that body temperature, pulse, blood pressure, respiration rate, and blood gas analysis were maintained at normal levels throughout sevoflurane exposure.

### Morris Water Maze Test

Twenty-four hours after anesthesia exposure, the spatial memory abilities were tested by using the Morris Water Maze (MWM). A circular, black painted pool (180 cm diameter, 50 cm deep) was filled with water to a depth of 30 cm and the water was made opaque by the addition of black non-toxic ink. The water temperature was maintained at 25±1°C. An invisible platform (10 cm diameter) was submerged 1 cm below the water line and placed in the centre of the northeast quadrant which was determined with four starting locations called north (N), east (E), south (S) and west (W) at equal distance on the rim. During four consecutive days, the rats were tested four times per day. Testing began by placing the rat at a random starting position, facing the pool wall, allowing them to swim and self discover the hidden platform. Rats who failed to locate the platform within 120 s would be guided gently to the platform. After arriving at the platform, the rat was allowed to stay on it for 30 s. The latency time to find the hidden platform was recorded and the average time from 4 trials represented as the daily result for the rat. On the 5th day, the hidden platform was removed, and each rat was allowed to swim freely for 120 s. The number of times that the rat crossed over the previous platform site was recorded. Each animal’s path was tracked by a computerizing video system (Electric factory of Anhui, China).

### The Tissue Preparation

After the Morris water maze test, all rats were anaesthetized by intraperitoneal injection of a lethal dose of Nembutal. The rats were perfused transcardially with 200 ml normal saline. Then, the rats’ hippocampus was quickly dissected. Immersion fixation was completed on tissues about 1 mm^3^ from the hippocampus of three rats per group for TEM observation. The brains of six animals per group were fixed in 10% formaldehyde, routinely dehydrated, and paraffin embedded for immunohistochemistry and TUNEL assay. The rest six rat’s hippocampus per group were frozen immediately on dry ice then stored at –80°C for RT-PCR analysis.

### Transmission Electron Microscopy Observation

The hippocampus, including dentate gyrus (DG) area, of each group’s rat was taken out immediately. Immersion fixation was completed on tissues about 1 mm^3^ from the hippocampus. Samples were rinsed in cold phosphate-buffered saline (PBS) and placed in 2.5% glutaraldehyde at 4°C for 4 h. The tissue was rinsed in buffer and post-fixed with 1% osmium tetroxide for 1 h. Then tissue was rinsed with distilled water before undergoing a graded ethanol dehydration series and was infiltrated using a mixture of one-half propylene oxide and one-half resin overnight. Twenty-four hours later, the tissue was embedded in resin. 120 nm sections were cut and stained with 4% uranyl acetate for 20 min and 0.5% lead citrate for 5 min. Ultrastructures of the nucleus, chondriosome, ribosome and synapse in hippocampus were observed by a transmission electron microscope (Phliphs Tecnai 10, Holland).

### Immunohistochemistry Assay

The tissues were embedded in paraffin, and transverse paraffin sections (4 mm thick) were mounted in silane-coated slides. For immunohistochemistry, the mounted sections were washed in 0.01 M PBS containing 0.3% Triton X-100 (pH 7.4, PBS-T), then immersed in 2% normal horse serum in PBS for 120 min at 37°C, incubated overnight at 4°C with CHOP antibody or Caspase-12 antibody (1∶200, Santa Cruz Biotechnology, USA) in PBS containing 1% bovine serum albumin, washed in PBS (3×5 min), incubated in biotinylated horse-anti-mouse IgG (1∶200, Boster Biotechnology, China) in PBS for 2 h at room temperature, washed in PBS-T (3×5 min), incubated in avidin–biotin–peroxidase complex solution (ABC, 1∶100, Boster Biotechnology, China) for 2 h at room temperature, then rinsed again in PBS-T (3×5 min). After staining, the sections were counterstained with hematoxylin. The sections were then dehydrated through ethanol and xylene before coverslips with Permount. Visualization was made by incubating the tissue for 10 min in 0.04% 3, 3-diaminobenzidine (DAB, Sigma Corporation, USA) containing 0.01% H_2_O_2_. Rat immunoglobulin IgG (1∶200, Biomeda Corporation, USA) was used instead of primary antibody as a negative control.

To assess the immunostaining quantification, We counted the number of CHOP and Caspase-12-positive cells in the same area, and examined the optical density of each slice in the both groups by using UTHSCSA Image Tools 3.0 (University of Texas Medical School, USA).

### Realtime-Reverse Transcription-quantitative PCR

For total RNA isolation, RNA was extracted from the specimens using the Trizol reagent kit (Invitrogen, USA) according to manufacturer’s protocol. For reverse transcription, RNA concentration was measured spectrophotometrically and 2 µg total RNA was added to the cDNA synthesis reaction system (20 µl) on a FTC2000 (Funglyn, Canada). The reaction mixture consisted of 4 µl 5× RT-Buffer, 2.5 µmol/L oligd (T), 5 mmol/L dNTPs and 20 U RNAasin (RNase inhibitor). The hexamers were annealed by incubating the samples to 70°C for 5 min. M-MLV reverse transcriptase 200 U (Promega, USA) was added, then incubated at 42°C for 60 min. The reaction was stopped by heating to 72°C for 10 min. For rt-PCR, the reaction mixture (40 µl) consisted of 4 µl cDNA, 35.2 µl SYBR® Premix Ex Taq™ (TaKaRa, China), 0.5 µl 5UTaq DNA polymerase and 0.3 µl of 20 pmol/µL CHOP or caspase-12 primer (Invitrogen, USA). The cDNA was denatured by heating to 94°C for 3 min. The template was amplified by 40 rounds of PCR (denaturation at 94°C for 10 s, annealing at 60°C for 30 s, extension at 72°C for 30 s) before measuring fluorescence at 72°C. The primers of CHOP as forward: 5′-CGGAGTGTACCCAGCACCATCA-3′, reverse: 5′-CCCTCTCCTTTGGTCTACCCTCA-3′, and Caspase-12 forward: 5′-AGGGATAGCCACTGCTGATACAGA-3′, reverse: 5′-CTGTCTCCACATGGGCCTTTGTT-3′, and GAPDH forward: 5′-GGTGGACCTCATGGCCTACAT-3′, reverse: 5′-GCCTCTCTCTTGCTCTCAGTATCCT-3′. The endpoint used in the real-time PCR quantification is defined as the PCR cycle number that crosses the signal threshold (Ct). The quantification of target gene expression was performed using the comparative Ct method, [20] and reported as the fold difference relative to the housekeeping gene.

### TUNEL Assay

Terminal deoxynucleotidyl transferase-mediated dUTP nick end labeling (TUNEL) assay was done to identify the extent of DNA fragmentation. The procedure was carried out using a commercial kit as instructed by the manufacturer (Kaiji, Nanjing, China). Briefly, after deparaffinization, the sections were treated with 20 µg/ml proteinase K for 10 min. After treatment with 0.3% H_2_O_2_ in methanol for 10 min, the sections were incubated with the TUNEL reaction mixture for 60 min at 37°C. Further incubation with peroxidase-conjugated antibody was performed for 30 min at 37°C. The sections were stained with diaminobenzidine solution for 10 min at room temperature and then counterstained with hematoxylin. The nuclei of apoptotic cells were labelled brown under DAB staining. The number of TUNEL positive cells and the total number of cells in the hippocampus were counted under 400× magnifications.

### Statistical Analysis

All statistical analysis was done with SPSS software, version 13.0 (SPSS Inc., Chicago, IL, USA). Group differences in the escape latency in the Morris water maze training task were analysed using one-way ANOVA with repeated measures, the factors being treatment and training day. The number and optical density of CHOP and Caspase-12-positive neurons, and the number of TUNEL positive neurons in the same area of hippocampus in six rats of both groups were examined. The data were analysed using the unpaired Student’s *t*-test if they were normally distributed (Kolmo-gorov-Smirnov test), otherwise the Mann-Whitney U test was used. Data are expressed as mean ± SD and a *P* value <0.05 was considered statistically significant.

## Results

### The Vital Signs and Arterial Blood Gas during Anesthetics Treatment

During the sevoflurane anesthesia, the vital signs were stable in rats, skin color was red, and there were no hypotension, respiratory depression and hypoxia occurred. There is no significant difference in the vital signs and arterial blood gas between two groups at the beginning (time 1), after 3 h (time 2) and the ending (time 3) (Table 1, *P*>0.05).

**Table 1:** The vital signs and arterial blood gas during anesthetics treatment.

	Groups	Time 1	Time 2	Time 3
HR(/min)	sham anesthesia	305.2±19.3	295.5±17.1	303.2±18.4
	sevoflurane anesthesia	310.9±20.2*	301.4±19.2*	298.6±19.9*
MAP(mmHg)	sham anesthesia	111.4±6.9	118.2±7.5	115.3±7.1
	sevoflurane anesthesia	113.5±7.3*	117.7±7.6*	110.8±7.2*
PaCO_2_(mmHg)	sham anesthesia	40.1±3.2	41.6±3.4	39.6±3.3
	sevoflurane anesthesia	40.4±3.3*	41.2±3.3*	40.2±3.6*
SPO_2_(%)	sham anesthesia	98.5±1.1	98.8±1.0	98.6±1.2
	sevoflurane anesthesia	98.7±1.2*	99.2±1.1*	98.4±1.1*

* *P*>0.05 *vs* sham anesthesia rats.

HR:heart rate, MAP: mean artery pressure, PaCO_2_: partial pressure of carbon dioxide in artery, SPO_2_: saturation of arterial blood oxygen.

### Sevoflurane Caused Spatial Memory Impairment in Aging Rats

**Figure 1:**
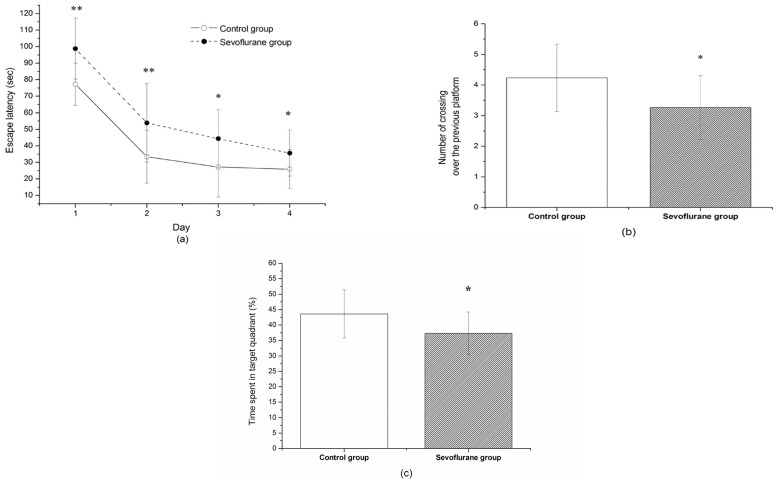
The effect of sevoflurane exposure on the memory and learning ability by Morris Water Maze testing. (a) Effects of sevoflurane on escape latency to find the hidden platform. The latency to find the hidden platform was significantly higher in sevoflurane-exposed rats than control rats. (b) The number of times that the rat crossed over the previous platform site within 120 s. (c) The time the rats stay in the target quadrant. Data are presented as mean ± SD. *P<0.05; **P<0.01, *vs* control group.

To evaluate the effect of sevoflurane exposure on the memory and learning ability, we subjected the rats to Morris Water Maze testing. As shown in Figure 1 a, all rats had a tendency of reduced latency to find the hidden platform as training progressed, indicating that the animals were learning from the day by day practice. However, we found that sevoflurane anesthesia had a significant effect on spatial orientation in the navigation task because it impaired the performance of the sevoflurane group. On the training day 1, 2, 3, and 4, the sevoflurane group rats showed significantly longer latency to locate the hidden platform than the control group (Figure 1 a), indicating significantly impairment in learning and memory functions after sevoflurane exposure. To check memory retrieval, the times that the animal crossed the platform position during the probe trial was analyzed. As shown in Figure 1 b, the number of times crossing over the previous platform site in the rats of the sevoflurane group were fewer than that of control group. Also the rats of the sevoflurane group spent less percentage of time swimming (25.3±2.6%) in the probe quadrant as compared with the control animals (34.1±3.5%) (*P*<0.05). These data suggested that the sevoflurane exposure could cause impairment in memory.

### TEM Observation

**Figure 2:**
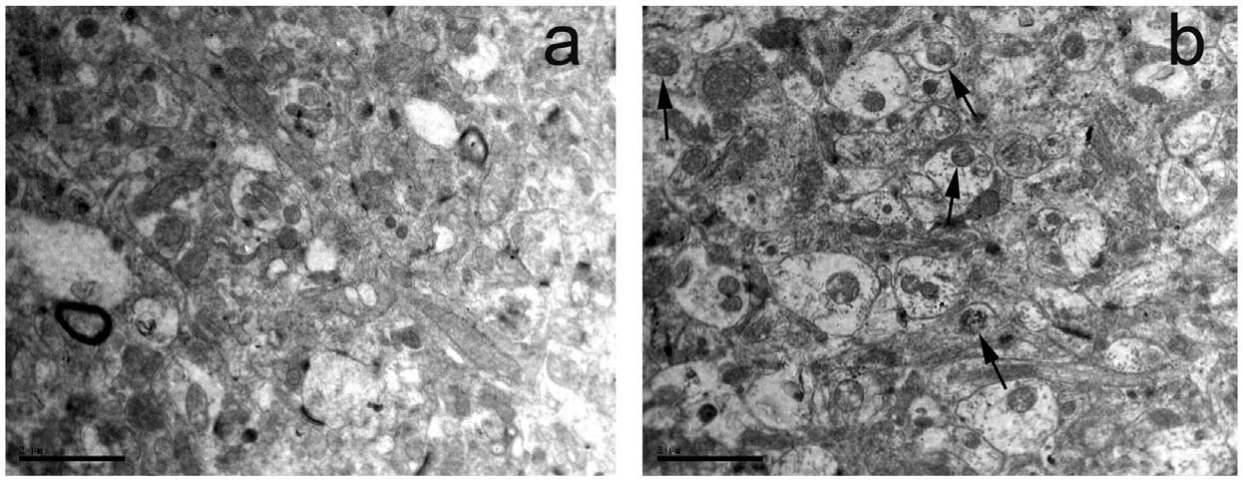
Ultrastructural changes of apoptotic neurons in sevoflurane group. These degenerating neurons showed an advanced stage of apoptosis, with chromatin clumping, condensation and margination (arrow), (a) a control rat. (b) a sevoflurane treated rat, Scale bar = 2 µm.

We examined the ultrastructure of neurons by TEM. The chromatin changes like clumping, margination and condensation were considered the most important evidence of apoptosis. In sevoflurane group, apoptotic neurons were condensed and had clumped chromatin with fragmentation of the nuclear membrane, verifying apoptotic degeneration (Figure 2).

### Up-regulation of CHOP and Caspase-12 in the Hippocampus of Aging Rats

**Figure 3:**
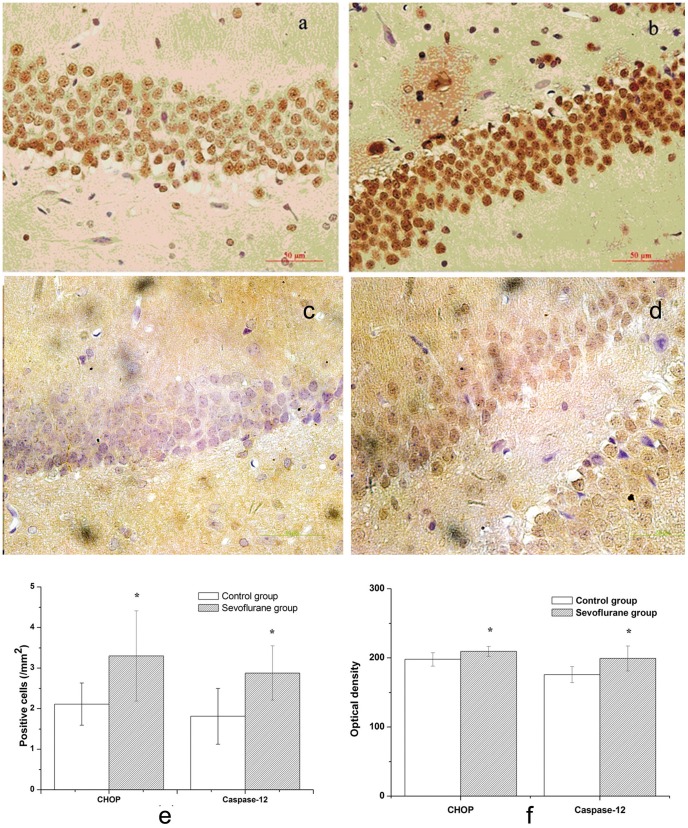
Immunohistochemistry of CHOP and Caspase-12 in hippocampus. (a) CHOP immunostain in control group ×400. (b) CHOP immunostain in sevoflurane group ×400. (c) Caspase-12 immunostain in control group ×400. (d) Caspase-12 immunostain in sevoflurane group ×400. (e) Numbers of CHOP and Caspase-12-positive cells. (f) Optical density of CHOP and Caspase-12-positive cells. Data are presented as mean ± SD. *P<0.05, *vs* the control group.

CHOP immunoreactivity was visualized in a granular immunostain pattern in the nucleus (Figure 3a and Figure 3b). Caspase-12 immunohistochemistry staining positive cells showed buffy granules with DAB staining in the cytoplasm (Figure 3c and Figure 3d). Compared to the control group, the sevoflurane group showed significant increases of immunostaining for CHOP and caspase-12 in the DG area of hippocampus (Figure 3, *P*<0.05).

**Figure 4:**
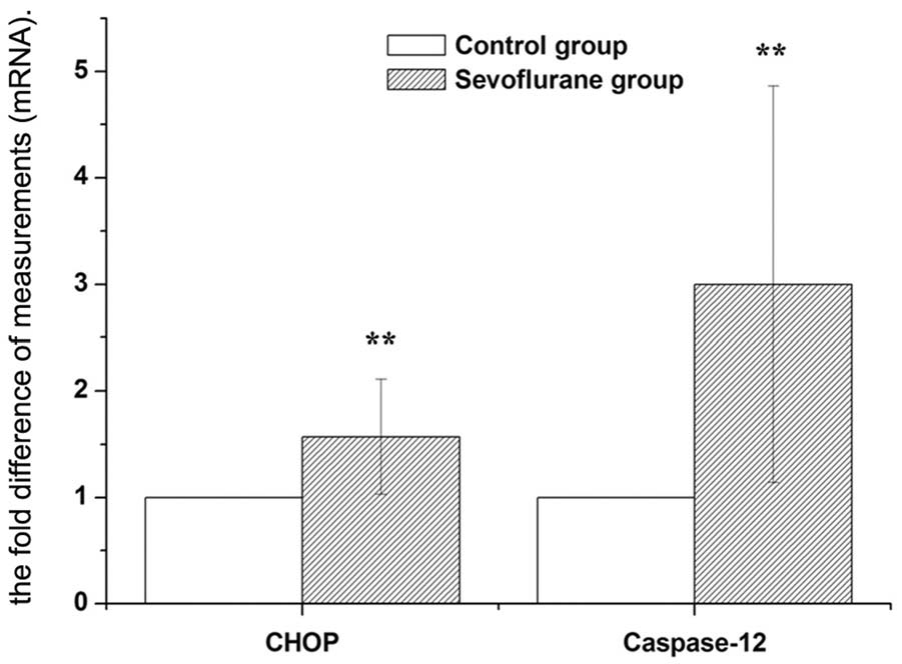
Expression of CHOP and Caspase-12 mRNA in hippocampus were analyzed by real-time RT-PCR. The real time reactions were performed in duplicates for both the target gene and GAPDH used as a housekeeping control. The relative expression was calculated using 2^−ΔΔCt^ method. Data are presented as mean ± SD. **P<0.01, *vs* control group.

Consistent with the alterations in CHOP and caspase-12 protein levels, we confirmed that CHOP and caspase-12 mRNA expressions were also up-regulated in the hippocampus of the sevoflurane-treated rats. As shown in the Figure 4, the expression of CHOP mRNA and caspase-12 were significantly higher in the sevoflurane group than that of the control group (1.57 folds and 3.0 folds respectively).

### Sevoflurane Induced Apoptosis in the Hippocampus of Aging Rats

**Figure 5:**
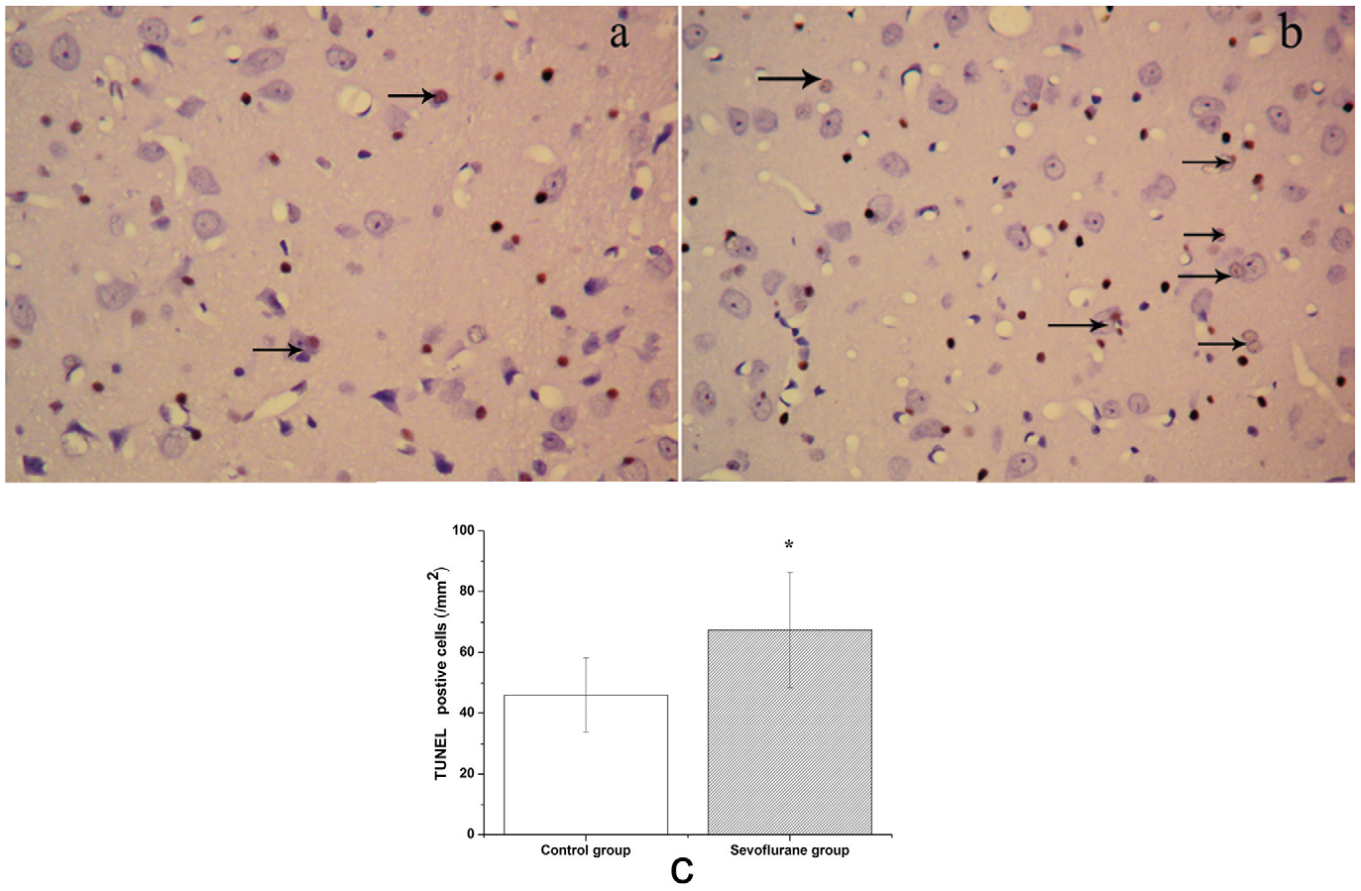
Sevoflurane induced apoptosis of cells in the hippocampus of aged rats. Apoptosis was examined by the TUNEL method. Upper: Photomicrographs of TUNEL-positive cells. (a) Control group. (b) Sevoflurane group. (c) Number of TUNEL-positive cells in each group. Data are presented as mean ± SD. *P<0.05, *vs* control group.

TUNEL is a common method for detecting DNA fragmentation that results from apoptotic signaling cascades. TUNEL immunohistochemistry revealed a distinctive pattern of nuclear staining (Figure 5). The number of TUNEL positive cells was significantly higher in the sevoflurane group compared to the control group in the hippocampal CA1 and DG region (Figure 5, *P*<0.05).

## Discussion

Previous studies have shown that volatile anesthetics, such as sevoflurane and isoflurane, are very helpful for reduction of perioperative mortality [21], [22]. But volatile anesthetics may also contribute to memory impairment by neurons lost in hippocampus through cells apoptosis [23], [24]. The Morris water maze test of the present study showed that the sevoflurane group rats showed significantly longer latency to locate the hidden platform than the control group on the training days. it suggested that sevoflurane anesthesia had a significant effect on spatial orientation in the navigation task because it impaired the performance of the sevoflurane group. Meanwhile the number of times that crossing over the previous platform site and the percentage of time swimming in the rats of the sevoflurane group are also decreased that indicating the impairment in memory [25]. The present data also demonstrated that 2% sevoflurane concentration for 5 h exposure would cause neurons apoptosis as the clumped chromatin with fragmentation of the nuclear membrane, verifying apoptotic degeneration under TEM observation. TUNEL staining revealed the same trend that the number of TUNEL positive cells was significantly higher in the sevoflurane group in the hippocampal CA1 and DG region. However, the upstream mechanism of volatile anesthetics induced apoptosis remains unknown.

The two most well studied pathways are the cell surface death receptor pathway and the mitochondria-initiated pathway [26]. The study showed that isoflurane might induce caspase activation and apoptosis through the mitochondrial pathway [27]. ER stress-induced apoptosis became an important pathological event in some neurological disease processes and neuronal cell death [8], [9], [28]. It has been reported that CHOP and caspase-12 are both key mediators of ER stress-induced apoptosis [29], [30]. CHOP is expressed at very low levels under physiological conditions, but strongly induced in response to ER stress [31]. Caspase-12 is localized specifically on the cytoplasmic side of the ER and is thought to play a pivotal role in ER stress-induced apoptosis [32]. CHOP activation occurs concomitantly with the activation of caspase-12, and activated caspase-12 in turn produces activation of the caspase cascade, then caspase-12 activates caspase-9, which in turn activates caspase-3, leading to cell death [11], [33]. In the present study, we found that 2% sevoflurane of 5 h exposure could increase the mRNA levels of CHOP and caspase-12, leading to up-regulation of the protein expression of CHOP and caspase-12 in the hippocampus of aging rats and accompany with the increasing TUNEL-positive cells. It suggested that the sevoflurane exposure may induce ER stress mediated apoptosis in the hippocampus, by increasing the expression of CHOP and caspase-12, then leading to neurons lost, and ultimately developed cognition impairment.

ER stress may be triggered by many disturbances, such as perturbed calcium homeostasis, oxidative stress, altered glycosylation levels, cholesterol overloading, and nutrient deprivation [32], [34]. The precise mechanisms underlying sevoflurane-induced ER stress are still elusive. One possible explanation is that volatile anesthetics alter cellular homeostasis of calcium in neurons. Volatile anesthetics have been shown to perturb calcium homeostasis by reducing calcium levels in ER and elevating calcium levels in cytosol and mitochondria [35]. The previous study revealed that inhalational anesthetics may induce cell damage by causing abnormal calcium release from the endoplasmic reticulum via excessive activation of IP3 receptors [35]. Another possible explanation is that volatile anesthetics trigger oxidative stress in neurons. Some previous studies showed that sevoflurane is capable of promoting the formation of reactive oxygen species (ROS) and perturbing redox status in vitro and in vivo [36], [37]. In our study, the neuronal cells apoptosis in the hippocampus of aging rats under sevoflurane exposure was detected, additionally the mRNA and protein levels of CHOP and caspase-12 were also increased. It may be the one mechanisms of the sevoflurane-induced neuron apoptosis. Therefore the further research is still need to do about dose and time effect.

In conclusion, the present study demonstrated that aging rats which exposure to sevoflurane could lead to neuronal degeneration and memory impairment. Our results suggest that sevoflurane can induce apoptosis through the ER stress pathway, which have identified at least partially the molecular mechanism by which sevoflurane induces apoptosis. Postoperative memory decline in the elderly has emerged as a major health concern [17]. It is important to study the mechanism of volatile anesthetic-mediated neurotoxicity in the aging patients for designing the safer anesthetics and preventing any toxic consequences by treating with ER stress antagonists.
